# Internet-based therapy for social anxiety in adults in Portugal (PORTiSOFIE) and its impact on sexual and relationship satisfaction: study protocol for a randomized controlled trial

**DOI:** 10.1186/s13063-025-09103-2

**Published:** 2025-11-06

**Authors:** Edna M. Martins, Patrícia M. Pascoal, Marco Pereira, Gerhard Andersson

**Affiliations:** 1 HEI-Lab: Digital Human-Environment Interaction Labs, Lusófona University, Lisbon, Portugal; 2 Clínica Universitária de Psiquiatria e Psicologia Médica, Faculdade de Medicina, Universidade de Lisboa, Lisbon, Portugal; 3 PSYLAB, Instituto de Saúde Ambiental (ISAMB), Faculdade de Medicina, Universidade de Lisboa, Lisbon, Portugal; 4 Center for Research in Neuropsychology and Cognitive and Behavioral Intervention, Faculty of Psychology and Educational Sciences, University of Coimbra, Coimbra, Portugal; 5 Department of Behavioural Sciences and Learning, Linköping University, Linköping, Sweden

**Keywords:** Internet-based intervention, Social anxiety, Sexual satisfaction, Relationship satisfaction

## Abstract

**Background:**

Social anxiety disorder (SAD) is a common condition that affects intrapersonal (e.g., distress and impairment) and interpersonal functioning (e.g., difficulties in amorous relationships). People with SAD tend to have difficulties in communicating feelings to partners and report lower levels of sexual and relationship satisfaction. Cognitive behavioral therapy (CBT) for SAD has been found to reduce symptoms of social anxiety, but less is known about its impact on relationship functioning. Internet-based CBT (iCBT) has gained attention for its primary advantage: bridging distances and saving time for the patient. In the planned study, we aim to translate and culturally adapt an iCBT program for social anxiety for use in Portugal (PORTiSOFIE) and measure the effects of the program on social anxiety symptoms. We will also study the impact on sexual and relationship satisfaction, as well as other relationship- relevant outcomes.

**Methods:**

We will conduct a randomized controlled trial and divide participants in a two-arm study with parallel groups (i.e., intervention group and attention group). After allocation, the intervention group will receive treatment, while the control group (i.e., attention group) will receive treatment 9 weeks later. The treatment is based on the Clark and Wells model of social phobia and consists of nine modules, with one module completed each week. Participants will answer questionnaires to assess social anxiety symptoms and relationship outcomes at pre-treatment, post-treatment, and 6-month follow-up. Participants’ partners will also be asked to complete questionnaires on the same periods of time, but only regarding relationship outcomes.

**Discussion:**

iCBT for SAD in adults has not yet been tested in Portugal, and overall, there is a gap in the literature regarding the role of partners in iCBT for SAD. We hope our study contributes to increasing accessibility to therapy (and better mental health) by promoting online evidence-based therapies. In addition, we hope it helps to better understand how relationship components are affected and affect clinical symptoms and their progression within receiving treatment.

**Trial registration:**

ClinicalTrials.gov NCT06767878. Registered on January 10, 2025.

## Administrative information

Note: the numbers in curly brackets in this protocol refer to SPIRIT checklist item numbers. The order of the items has been modified to group similar items (see http://www.equator-network.org/reporting-guidelines/spirit-2013-statement-defining-standard-protocol-items-for-clinical-trials/).

**Table:** 

Title {1}	Internet-based therapy for social anxiety in adults in Portugal (PORTiSOFIE) and its impact on sexual and relationship satisfaction: Study protocol for a randomized controlled trial.
Trial registration {2a and 2b}.	ClinicalTrials.gov Identifier: NCT06767878 https://clinicaltrials.gov/study/NCT06767878
Protocol version {3}	Protocol version 1.0, 2025–01-30
Funding {4}	This work is financed by national funds through FCT—*Fundação para a Ciência e a Tecnologia, I.P.,* under project 2024.03961.BD.
Author details {5a}	Edna M. Martins, HEI-Lab: Digital Human–Environment Interaction Labs, Lusófona University, Lisbon, Portugal, edna.martins@ulusofona.pt (Corresponding author) Patrícia M. Pascoal, 1. HEI-Lab: Digital Human–Environment Interaction Labs, Lusófona University, Lisbon, Portugal 2. Clínica Universitária de Psiquiatria e Psicologia Médica, Faculdade de Medicina, Universidade de Lisboa, Lisbon, Portugal, 3. PSYLAB, Instituto de Saúde Ambiental (ISAMB), Faculdade de Medicina, Universidade de Lisboa, Lisbon, Portugal Marco Pereira, University of Coimbra, Center for Research in Neuropsychology and Cognitive and Behavioral Intervention, Faculty of Psychology and Educational Sciences, Coimbra, Portugal Gerhard Andersson, 1. HEI-Lab: Digital Human–Environment Interaction Labs, Lusófona University, Lisbon, Portugal, 2. Linköping University, Department of Behavioural Sciences and Learning, Linköping, Sweden
Name and contact information for the trial sponsor {5b}	FCT—*Fundação para a Ciência e a Tecnologia, I.P.* Email adress: bolsas@fct.pt
Role of sponsor {5c}	Sponsor and funder have no role in the study design, collection, management, analysis, and interpretation of the data and writing of the report. Investigators have full control of decision to submit the report for publication.

## Introduction

### Background and rationale {6a}

Social anxiety disorder (SAD), according to the DSM-5 [1] is defined by an emotional and/or behavioral pattern marked by intense fear, which arises from a real or imagined possibility of being negatively evaluated by others. Individuals with SAD usually have difficulties in interpersonal contexts, which affects the quality of their relationships, including amorous relationships. For example, there is a tendency for these individuals to have poor communication skills which, in turn, can have an impact on emotional self-disclosure with romantic partners [2]. This can compromise the intimacy experienced in the relationship [3] and negatively affect relationship and sexual satisfaction [4, 5], which are strongly interrelated constructs [6]. Research suggests that difficulties in emotional regulation (both intra and inter-personal) are present in individuals with SAD, due to specific characteristics found within the disorder (e.g., bias processing negative information) [7] and that those difficulties can impact amorous relationships (e.g., relationship satisfaction) [8]. Theoretical models, such as the interdependence theory [9], state that people influence one another’s experiences, highlighting the importance of considering dyadic influences on emotions, behaviors, and thoughts. However, existing studies have not focused on the impact of psychological treatments for SAD on the relational dimensions of the amorous relationships of these individuals and/or their partners, thus leaving a gap regarding treatment effects on interpersonal variables typically affected by SAD. Accordingly, the present study will first translate and culturally adapt an internet-based cognitive behavioral therapy (iCBT) program for social anxiety for a Portuguese population. Secondly, it will evaluate its effect on sexual and relationship satisfaction and other relationship related variables (i.e., dyadic adjustment, closeness, emotion regulation and communication), in adults in monogamous amorous relationships.

Cognitive behavioral therapy (CBT) is considered the first-line psychological intervention for SAD [10], with its efficacy proven in several randomized controlled trials [RCT] [11]. ICBT for SAD was initially developed in Sweden and has since been tested in many countries and settings (e.g., Australia, Switzerland, UK) [12].

Meta-analysis of studies on iCBT for SAD indicates equivalent overall effects as face-to-face treatments [13]. In addition, the online delivery does not jeopardize therapeutic gains or the effectiveness of treatment, which is particularly important given that individuals with SAD tend to avoid seeking help because it requires direct contact with third parties (e.g., therapist, receptionist). As such, the online modality aids treatment adherence [12] and increases accessibility to therapy [14]. In Portugal, there is a lack of online therapy services, and there are limitations that need to be overcome relating to resistance to online therapy, such as a lack of knowledge and training among therapists [15]. We hope that this study will contribute to finding solutions for those seeking online therapy, both patients and therapists, and open doors for those who are not yet familiar with the modality. Even so, in Portugal, iCBT therapy for social anxiety in adolescents has already been implemented and has shown preliminary efficacy [16]. However, there is still a gap regarding the efficacy of iCBT for SAD in adults. Moreover, social anxiety impacts interpersonal functioning. It is therefore logical that research moves towards finding what interpersonal variables (such as sexual and relationship satisfaction, dyadic adjustment, closeness, emotion regulation and communication) should be targeted to improve clinical results. Targeting such variables could benefit both those who suffer from this disorder and those who are affected by it indirectly (e.g., spouses/partners).

### Objectives {7}

The present study aims to adapt and test the efficacy of an iCBT program for adults, namely, the iSOFIE (*The Internet Social Phobia*) [17], a self-help treatment informed by the model of SAD by Clark and Wells [18]. The iSOFIE program has been tested in many trials in Sweden, as well as in other countries like Norway and Romania, where it was found to be effective [19, 20]. Since it was originally developed in Swedish, our first goal concerning this project is to translate and culturally adapt it to European Portuguese, thus originating the PORTiSOFIE (*The Portuguese Internet Social Phobia*).

Our second goal is to test the effect of PORTiSOFIE on the following outcomes:

- Primary outcomes (SAD symptoms, relationship and sexual satisfaction);
- Secondary outcomes (dyadic adjustment, closeness, emotion regulation and communication).

For this, we will conduct a controlled study, with the following aims:

1. Dissemination of study and recruitment of potential users;
2. Implementation of PORTiSOFIE and measurement of relevant outcomes;
  - Allocate participants to the groups (intervention vs. attention control group) and assess primary outcomes (i.e., social anxiety symptoms, sexual satisfaction and relationship satisfaction) and secondary outcomes (i.e., dyadic adjustment, closeness, difficulties in interpersonal emotion regulation, and communication) at baseline/pre-treatment;
  - Implementation of PORTiSOFIE with a weekly assessment of the primary outcomes;
  - Assess of primary and secondary outcomes at posttreatment and 6-month follow-up.

The study will expand knowledge on the use of iCBT for adults with SAD in a new cultural context andlanguage. Considering that amorous relationships are usually negatively affected by SAD, we also aim to highlight the role of partners by incorporating relationship dimensions (dyadic adjustment, closeness, emotion regulation and communication). This approach will help to further understand the effect of the intervention on an important interpersonal context.

### Trial design {8}

The present study will be an RCT with a post-intervention follow-up at 6 months after completed treatment. Following recruitment, included participants will be divided into two groups through a “blinded” randomization process (1:1 ratio). The trial is parallel, two-armed, and open. One group will be the intervention group (i.e., treatment) and the other will be the control group (i.e., attention).

## Methods: participants, interventions, and outcomes

### Study setting {9}

Participants will be recruited from the general public in Portugal via social media. Telephone interviews, after screening questionnaires are completed, will be conducted to assess eligibility to participate. The study will be completed entirely online with no face-to-face meetings. 

### Eligibility criteria {10}

People with clinical and subclinical symptoms of social anxiety will be included. Scores on the screening questionnaires indicating at least moderate levels of social anxiety will be the first inclusion criteria. In addition, participants must be aged 18 years or over, be fluent in Portuguese, have access to a device with internet, be in a monogamous relationship (regardless of sexual orientation), and not be receiving any ongoing treatment for social anxiety (e.g., stable antidepressants). The criterion of being in a monogamous relationship was chosen to reduce variability in relational dynamics that could affect the interpretation of outcomes related to relationship dimensions. Including participants in monogamous relationships allows for a more consistent assessment of interpersonal variables across the sample. However, we acknowledge that this criterion may limit the generalizability of the findings to individuals in non-monogamous or alternative relationship structures. Future research should consider including more diverse relationship types to enhance external validity.

The exclusion criteria include self-reported severe psychiatric disorders such as psychosis or personality disorder. Planned or ongoing medical treatments (e.g., surgery) and other personal circumstances that will interfere with the treatment (e.g., planned travel abroad) will also be exclusion criteria.

.

Those who meet the initial inclusion criteria will subsequently be contacted for a telephone interview, which will be carried out by an experienced clinician, with the aim of assessing social anxiety based on the SCID-5.

During the interview we will also mention the importance of having the partners participating (not to receive intervention, but to fill out questionnaires to assess relationship dimensions). Partner participation can only be validated after the partner itself accepts the informed consent and completes sociodemographic and screening questionnaires. The inclusion criteria for partners will be: age 18 years or over, fluency in Portuguese, and access to a device with internet. Participants who need psychological support will be referred for clinical services for treatment.

### Who will take informed consent? {26a}

Interested people must enter a link to access the study. They will be directed to a webpage that will contain an informed consent, sociodemographic, and screening questionnaires. The same process will occur for partner eligibility. After the screening is completed, eligible participants will be contacted for a telephone interview that will determine if they and their partners are going to be included in the trial.

### Additional consent provisions for collection and use of participant data and biological specimens {26b}

No biological specimens will be collected from this study.

## Interventions

### Explanation for the choice of comparators {6b}

Participants in the intervention group will receive PORTiSOFIE treatment. If the intervention in our study shows preliminary efficacy, it will be offered to the control group after the study period.

While the intervention group is undergoing treatment, participants in the wait-list control group (attention group) will be monitored weekly via the iTherapy platform. Each week, participants will be asked to complete a brief symptom self-report instrument focusing on key indicators of social anxiety. The therapist will review the responses and may follow up with a short message or contact to acknowledge receipt and check on general well-being. No therapeutic content or guidance will be provided during this monitoring, in order to preserve the integrity of the control condition while ensuring ethical oversight and participant safety.

To support retention, both groups will receive automated email reminders at important points in the study (e.g., before scheduled assessments or symptom check-ins). These messages will serve to reinforce participant engagement, clarify upcoming steps, and help reduce attrition. Additionally, the weekly contact with a therapist in the control group is designed not only for monitoring purposes but also to foster a sense of continued involvement, reducing the likelihood of dropout.

### Intervention description {11a}

The study will occur in the iTherapy platform (this is a secure platform on which several online studies have already been conducted [18]), where we will set up the intervention. After allocation to the treatment or attention group, an online therapist will be assigned to each of the participants and will provide asynchronous support (e.g., feedback, clarification of doubts) throughout the study. The protocol has minimal guidance, and the therapist actions are limited to interactions on the platform via a messenger portal. As the therapist will have limited contact with participants with only text support, we anticipate having one therapist (the principal investigator of the project) providing this support. Partners will not have access to a therapist as they will not receive intervention. The intervention lasts 9 weeks, with 1 module per week. Modules include:

1. Introduction of the program to the participant and overview of social anxiety disorder and symptoms;
2. Definition and role/function of negative automatic thoughts; and the model of social anxiety of Clark and Wells [15] is presented;
3. Cognitive distortions and strategies to challenge those distortions and negative thoughts;
4. Anticipation of difficulties that may arise when challenging thoughts, and psychoeducation about behavioral strategies to test those thoughts;
5. Exposure Introduction and challenge of participants to put it in practice through guided orientation;
6. Explanation of the concept of self-focus attention and of safety behaviors, and introduction of strategies to reduce those mechanisms;
7. Discussion and offered solutions to difficulties that may have raised during exposure/fearful situations;
8. Social skill training (e.g., ability to communicate assertively, active listening others, dyadic communication);
9. Information about relapse prevention and gains maintenance.

Participants who experience significant emotional distress during the intervention, particularly those with more severe symptoms of SAD, will be explicitly encouraged to contact their personal therapist or seek professional support.

### Criteria for discontinuing or modifying allocated interventions {11b}

All participants have the right to withdraw from the study in any phase, with no consequences. If they wish to receive psychological support, investigators will reference them for clinical treatment.

### Strategies to improve adherence to interventions {11c}

To improve adherence to treatment, individual guidance will be available from the therapist who will respond to specific doubts and difficulties encountered during the study.

### Relevant concomitant care permitted or prohibited during the trial {11d}

Participants who are taking medication or receiving other forms of treatment for social anxiety are not eligible for participation in the trial. Also, those who self-report in the sociodemographic questionnaire or during the telephone interview as having psychosis or personality disorder will be excluded.

### Provisions for post-trial care {30}

The intervention that will be offered has shown to be effective in reducing social anxiety symptoms in other countries, so we do not expect it to include any risks of harm for participants.

### Outcomes {12}

Primary outcomes include social anxiety symptoms and sexual and relationship satisfaction.

### Primary outcomes

Social anxiety symptoms:

- Liebowitz Social Anxiety Scale for Adults (LSAS) [21] has 24 items, responded in a 4-point scale. Each item is rated regarding anxiety and avoidance, and so the total score of the scale ranges between 0 and 144, with higher scores indicating higher levels of social anxiety (i.e., below 40 – no discomfort; 40–55 – Mild social discomfort; 55–65 – Moderate social phobia; 65–80 – Marked social phobia; 80–95 – Severe social phobia; Above 95 – Very severe social phobia).
- Social Phobia Inventory (SPIN) [22] is composed by three subscales: fear, avoidance, and physical discomfort. It contains 17 items in total (16 in the Portuguese version), assessing the symptoms corresponding to these domains. The scale is responded on a 4-point scale, and total score is between 0 and 68, with higher scores indicating higher levels of social anxiety.

Relationship satisfaction:

- Global Measure of Relationship Satisfaction (GMREL) [23] is constituted by 5 items answered in a 7-point Likert scale. Scores range between 5 (low relationship satisfaction) and 35 (high relationship satisfaction).

Sexual satisfaction:

- Global Measure of Sexual Satisfaction (GMSEX) [23] has 5 items responded in a 7-point Likert scale, with total scores ranging from 5 to 25, with higher values indicating greater sexual satisfaction.

### Secondary outcomes

Dyadic adjustment:

- Revised Dyadic Adjustment Scale (RDAS) [24], with three subscales: consensus, satisfaction, and cohesion. It is a 14-item measure that asks people to rate aspects of the relationship on a 5- or 6-point response scale. Total scores range between 0 and 69, with higher values denoting greater dyadic adjustment.

Closeness

- Inclusion of Other in the Self Scale (IOS) [25], a single-item pictorial measure that assesses how close the respondent feels with another person (in this case the amorous partner).

Emotion regulation

- Difficulties in Interpersonal Emotion Regulation (DIRE) [26], which has two subscales: venting and reassurance-seeking, constituted by a total of 24 items. Three hypothetic situations are presented, and for each one, the respondent must indicate how the situation is perceived on a scale from 0 (not at all distress) to 100 (extremely distressed). Then, for each of the scenarios, is asked the likelihood (i.e., 4-point Likert scale) of responding in 7 different presented ways. Higher scores indicate higher levels of difficulties in interpersonal emotion regulation strategies.

Intimacy (communication)

- Personal Assessment of Intimacy in Relationships (PAIR) [27] is constituted by 36 items, which are responded in a 5-point Likert scale (from 1 “strongly disagree” to 5 “strongly agree”), with 18 items being reversed scored, so that higher values correspond to higher levels of intimacy. For the present study we will only use the communication subscale with 10 items.

### Participant timeline {13} Fig. 1

**Fig. 1:**
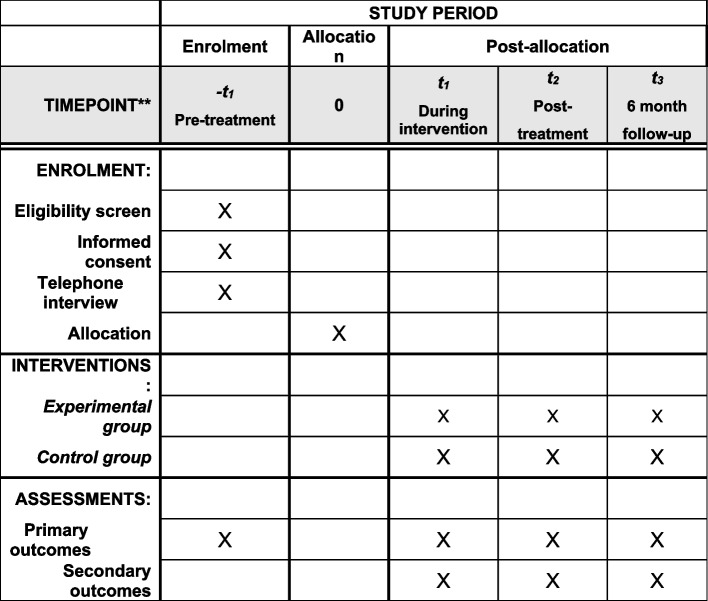
Overview of the study process

### Sample size {14}

The sample size calculation was based on the primary outcome of social anxiety as measured by the LSAS. To detect a statistically and clinically significant effect of the intervention, we assumed a large effect size (Cohen’s *d* = 0.80), consistent with prior meta-analytic findings on internet-delivered cognitive behavioral therapy (CBT) for social anxiety disorder. For example, Andrews et al. [28] reported a mean effect size of *g* = 0.80 for computerized CBT across anxiety and depression disorders, including social anxiety. Given that Hedges’ *g* and Cohen’s *d* are practically equivalent in larger samples, we considered this value appropriate for our sample size estimation. Based on this effect size and considering the planned analyses (e.g., intention-to-treat approach and ANCOVA for comparing pre- and post-intervention differences), we estimated that a total of 52 participants (26 per group) would be sufficient to achieve 80% power at a 5% significance level. However, to account for expected dropout rates in CBT studies (approximately 26%), we aimed to recruit at least 70 participants (35 per group). Among all primary outcomes considered, the chosen outcome required the largest sample size to detect a clinically meaningful effect and thus was used for this calculation.

### Recruitment {15}

The study will be disseminated through online platforms and will adhere to the ethical regulations. We will include people with clinical and subclinical expressions of social anxiety. Those who meet the inclusion criteria will subsequently be contacted for a telephone interview, and those who report symptoms of SAD will be considered suitable for inclusion in the study. During the interview, eligibility criteria will be verified and study procedures explained. At this stage, participants will also be encouraged to involve their romantic partner throughout the study. To support this, a brief educational summary will be provided, outlining the nature of social anxiety disorder, the goals of the intervention, and how partners can contribute positively to the participant’s progress and motivation. The summary will cover key topics such as the core symptoms of SAD (e.g., fear of negative evaluation, avoidance), its potential impact on amorous relationships (e.g., reduced intimacy, problems in communication), and practical ways in which partners can offer support, such as encouraging gradual exposure, maintaining open and empathetic communication, and avoiding reinforcing avoidance behaviors. It will also include basic guidance on partner self-care and how to support treatment adherence in a constructive and collaborative manner.

## Assignment of interventions: allocation

### Sequence generation {16a}

After participant and partner selection, we will allocate participants to intervention and control groups in a 1:1 ratio. This allocation will be carried out through a randomization sequence in statistical program R.

### Concealment mechanism {16b}

Randomization will be performed by an external person not involved in the study. 

### Implementation {16c}

**Fig. 2:**
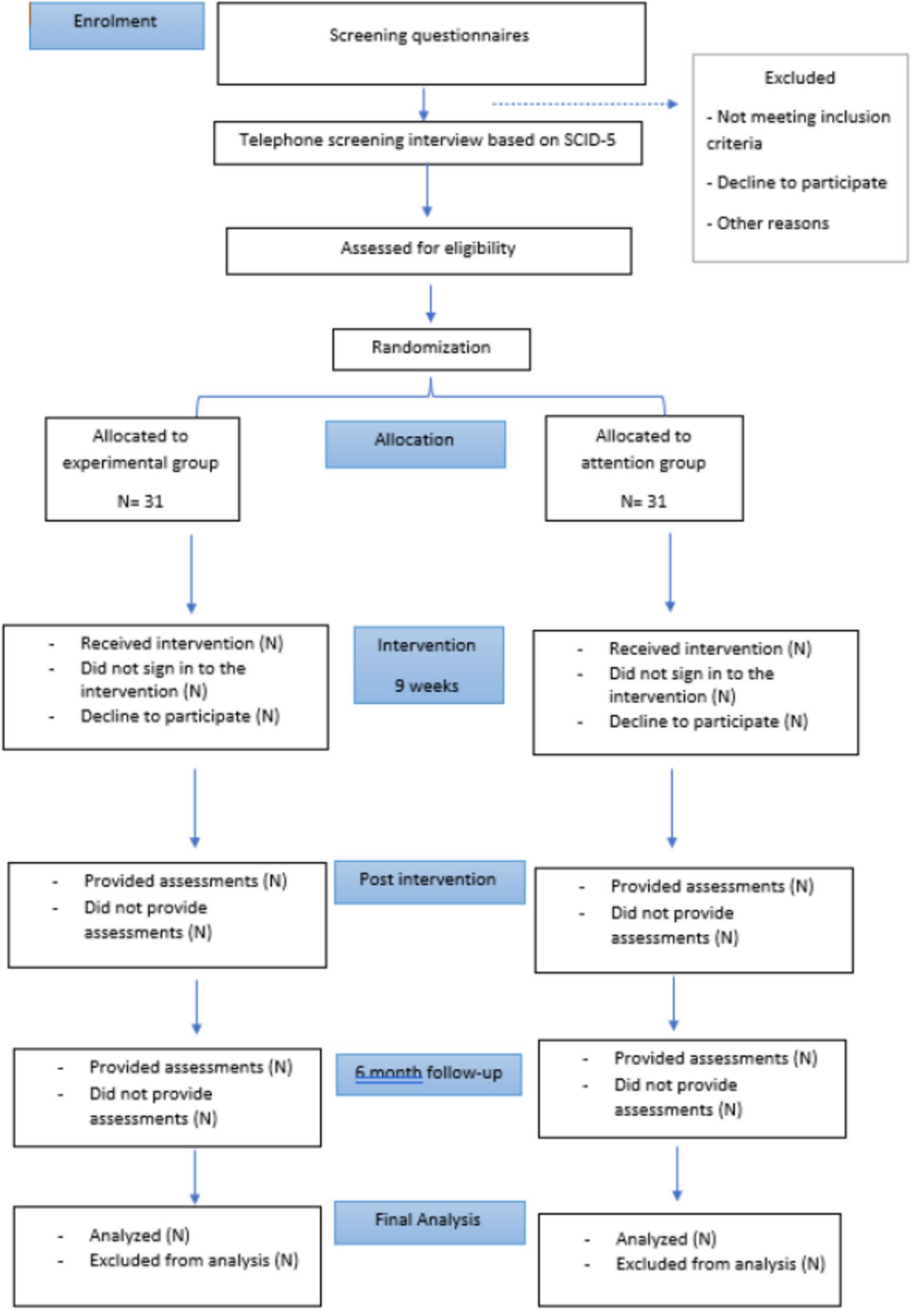
Study Flow Chart

Since randomization is automated and based on study code, the role of the independent person will only be to generate the sequence. As this is a digital study, most will be done online but coordinated by a researcher. Participants will be enrolled and assigned to different groups. Fig. 2.

## Assignment of interventions: blinding

### Who will be blinded {17a}

As this is a psychological intervention study, blinding of participants and the online therapist is not feasible. This may introduce performance or expectancy biases, particularly in self-reported outcomes. To mitigate these risks, both groups will receive structured content and regular contact through the platform, reducing differences in attention. As the data analysis will be conducted by members of the research team who are also involved in participant support, blinding during statistical analyses is not possible. However, analyses will be based on pre-specified hypotheses and outcome measures to reduce bias. All procedures and decisions will be transparently reported to enhance the credibility of the findings.

### Procedure for unblinding if needed {17b}

Not relevant.

## Data collection and management

### Plans for assessment and collection of outcomes {18a}

Participants will complete primary outcomes (i.e., social anxiety symptoms, relationship, and sexual satisfaction) and secondary outcomes (i.e., dyadic adjustment, closeness, emotion regulation and communication) at pre-treatment, post-treatment, and at 6-month follow-up. In addition, during the intervention, there will be weekly assessments of primary outcomes. Participants’ partners will also complete measures of primary and secondary interpersonal outcomes (relationship satisfaction, sexual satisfaction, dyadic adjustment, relationship closeness, difficulties in interpersonal emotion regulation, and communication) at pre-treatment, post-treatment, and 6-month follow-up.

### Plans to promote participant retention and complete follow-up {18b}

We expect some difficulties associated with sample collection and retention rates during the implementation of the protocol. We will collect a number of participants above the minimum needed, so that dropout does not decrease statistical power. To minimize dropout, participants and their partners will receive encouragement to complete the assessments at each stage. For participants, therapist feedback will also serve to promote continued engagement. For partners, we will provide a short and clear explanation of their role and its relevance to the study. We also anticipate that difficulties in recruitment and adherence of partners may emerge. If this happens, we will consider excluding dyadic analysis but maintaining the analysis of interpersonal variables only.

### Data management {19}

To ensure data protection, all online data collected will be protected by password, and only investigators from the research team will be allowed to access the database that will be saved in an encrypted manner. To ensure confidentiality, all IPs saved will be deleted. Personal identification data will not be saved together with data collection online, as each participant will have a generated code.

### Confidentiality {27}

There will be an individual log-in for each participant and each partner, which protects the confidentiality of data. Participants will enter the platform with their credentials to access the therapy modules, as well as the questionnaires that are requested throughout the study. Partners also need to log in to the platform (with their individual credentials) to answer the questionnaires needed; however, they will not have access to the intervention modules.

### Plans for collection, laboratory evaluation, and storage of biological specimens for genetic or molecular analysis in this trial/future use {33}

Not applicable.

## Statistical methods

### Statistical methods for primary and secondary outcomes {20a}

Descriptive statistics will be reported using means and standard deviations for continuous variables, and frequencies for categorical variables. Primary analyses will follow the intention-to-treat principle. To assess treatment effects, we will conduct analyses of covariance (ANCOVA) comparing post-intervention scores between the experimental and control groups, while adjusting for baseline levels of the respective outcome variable (e.g., LSAS for social anxiety), as well as for age and relationship duration, which will be included as covariates due to their known associations with treatment outcomes and interpersonal functioning. This approach allows for greater precision in estimating intervention effects and accounts for potential baseline imbalances. Missing data will be addressed using multiple imputation under the assumption of missing at random (MAR), in line with recommendations from meta-analytic research on dropout in CBT trials [29]. A two-tailed significance level of *p* < 0.05 will be used for all hypothesis testing. Effect sizes (Cohen’s *d*) will also be calculated to assess the magnitude of between-group differences, consistent with prior meta-analytic findings on the efficacy of internet-based CBT for social anxiety disorder [30].

### Interim analyses {21b}

No interim analyses are planned. Given the short duration of the intervention, the minimal risk involved, and the primary aim of evaluating preliminary efficacy, interim analyses were deemed unnecessary and potentially bias-inducing.

### Methods for additional analyses (e.g., subgroup analyses) {20b}

Considering that we aim to have participants and their respective partners responding, we might need to analyze subgroups such as the group of participants whose partner dropped out, the group of participants whose partner continued on the study until the follow-up, and others.

### Methods in analysis to handle protocol non-adherence and any statistical methods to handle missing data {20c}

To handle missing data, we will use multiple imputation.

### Plans to give access to the full protocol, participant level-data and statistical code {31c}

We will follow all ethical issues regarding confidentiality of participants in every phase of the study or its publication. After the clinical trial is completed, if the intervention shows to reduce SAD symptoms, we plan to give full access to the protocol, as we aim to disseminate the intervention to those who seek help.

#### Oversight and monitoring

### Composition of the coordinating center and trial steering committee {5d}

The study is being developed within Lusófona’s University, and it is associated with the research unit Digital Human–Environment Interaction Lab (HEI-Lab). HEI-Lab will be responsible for offering support in fundamental aspects of the execution of the project, namely through SPIC (Service of Psychology, Innovation and Knowledge), a clinical service with consultations that will be available for referencing participants (or partners) who need or want therapy. The setup of the intervention will be done on the iTherapy platform, which belongs to Linkoping University, and some tasks (e.g., review of protocol, and data analysis) will be developed in collaboration with the Center for Research in Neuropsychology and Cognitive Behavioral Intervention (CINEICC) of the University of Coimbra. All authors will participate in periodic meetings to discuss and align strategies for implementing and conducting the trial.

### Composition of the data monitoring committee, its role and reporting structure {21a}

We do not intend to have a Data Monitoring Committee, since it is a low-risk intervention, and it has been replicated with effectiveness in other countries. We will submit ethical reports to the ethics committee to ensure that all steps of the study follow the best care standards.

### Adverse event reporting and harms {22}

Any adverse or unintended effects will be directly reported to the main investigator and will be managed by the main investigator who is responsible for forwarding patients to clinical services (SPIC).

### Frequency and plans for auditing trial conduct {23}

We expect to meet monthly with at least the principal investigator and supervisor of the project being present; but, when possible, auditing of the trial will be performed with all investigators (online or face-to-face).

### Plans for communicating important protocol amendments to relevant parties (e.g. trial participants, ethical committees) {25}

Any modification of the study or the protocol will be communicated to all investigators, as well as to the ethical committee. 

### Dissemination plans {31a}

Results of the study will be published in peer-reviewed journals. Also, the investigators will present them in scientific meetings.

## Discussion

Internet-based interventions have several advantages for patients, such as cost-effectiveness, accessibility, and better time management. This study is an important step towards allowing people to choose which modality is more convenient for them (i.e., face-to-face or online). We hope that this study will actively contribute to the clinical development of online therapies and their accessibility to the population (especially the Portuguese population). The protocol we are going to test has been tested and shown to be effective in other European countries. We have already culturally adapted it to the Portuguese population, so we expect it to be effective in our country. However, assessing the impact of therapy on relationship dimensions is a new topic that has yet to be explored because it has been neglected in research, and so we hope to contribute with innovative knowledge.

This study has several limitations that should be considered. Due to the waitlist control design, participants are aware of their group allocation, which limits participant blinding. However, the therapist providing support interacts with participants only via asynchronous text messages through a secure platform and does not have face-to-face or video contact. This may reduce potential bias typically associated with non-blinded interventions.

Another anticipated challenge involves the recruitment and retention of partners. While the study is designed to capture relational outcomes from both members of the couple, some partners may not agree to participate or may drop out over time. If needed, we will compare outcomes between participants whose partners participated and those whose partners did not, although we cannot yet determine whether such comparisons will be feasible.

Lastly, although a 6-month follow-up is included to assess the maintenance of treatment gains, a longer-term follow-up (e.g., 12 months) is not planned due to time constraints related to the doctoral funding period. Future research should aim to assess the long-term impact of iCBT for social anxiety, as well as develop strategies to increase partner engagement and dyadic data completeness.

### Trial status

Protocol version 1, 2025–01–30, Recruitment did not yet commence. Recruitment is planned to begin in January 2026 and extend until December 2026.

## Data Availability

After the publication of trial results, a de-identified dataset will be available from the Principal Investigator upon a reasonable request.

## Abbreviations

APA: American Psychiatric Association
DSM-5: Diagnostic and Statistical Manual of Mental Disorders (Fifth Edition)
CBT: Cognitive behavioral therapy
SAD: Social anxiety disorder
iCBT: Internet-delivered cognitive behavioral therapy
RCT: Randomized controlled trial
SCID-5: The Structured Clinical Interview for DSM-5
iSOFIE: The Internet Social Phobia
PORTiSOFIE: The Portuguese Internet Social Phobia
HEI-Lab: Digital Human Environment Interaction Lab
SPIC: Service of Psychology, Innovation and Knowledge
CINEICC: Center for Research in Neuropsychology and Cognitive Behavioral Intervention
